# *Retrovirology*: 3 at age 2

**DOI:** 10.1186/1742-4690-3-30

**Published:** 2006-05-31

**Authors:** Kuan-Teh Jeang

**Affiliations:** 1 the National Institutes of Health, Bethesda, MD, USA

## Abstract

*Retrovirology *announces new editorial board members and reprises progress over the first two years of publishing.

## A word of thanks

As of this writing, *Retrovirology *has completed a little more than 24 months of continuous publishing. The journal has published 156 articles or roughly 6 articles per month, all in Open Access format. Since retrovirus research is a subsector of all virus research and *Retrovirology *considers only basic science papers, this rate of monthly publication seems reasonable at this juncture in journal development. I am also pleased with the quality of the papers that *Retrovirology *has published. To a large extent credit for this achievement must go to our Editorial Board members. Hence, I take this opportunity to thank departing members (Eric Arts, Steve Jacobson, Gerold Feuer, Kiyoshi Takatsuki, Romas Geleziunas, Mika Salminen, W.A. Paxton, Michel Tremblay, Naoki Yamamoto, Yoko Aida, Masataka Nakamura, Klaus Uberla, Walter Guenzberg, Myra McClure, Vanessa Hirsch, Vineet KewalRamani, Wendy Maury, Pierre Corbeau, Guido Vanham and Lee Ratner) who have served the journal ably over the last two years. In turn, I welcome new members who have joined *Retrovirology *(Michael Burkrinsky, Kathy Boris-Lawrie, David Derse, Juan Lama, Renaud Mahieux, Leonid Margolis, Rogier Sanders, James K. Hildreth, Toshiki Watanabe, Naoki Mori, Tatsuo Shioda, Ariberto Fassati, Tahir A. Rizvi, Janice Clement, Chris Aiken, Neil Almond, Stephen P. Goff, William Hall, Warner Greene, and Richard Zhao). The full list of current *Retrovirology *Editors and Editorial Board members can be viewed at .

## The reasons for Open Access

Undeniably, retrovirus researchers are well-served by several established subscription-based journals. However, on the current scientific landscape, there is a choice between two different ways of publishing research: the traditional journal (where subscribers and sometimes authors pay) and the Open Access model (where authors pay). The Open Access model embraces a novel concept that authors (or the funders of their research) pay for the submission and publication of papers. Once published, Open Access articles are free in full text to all interested readers. This means that your scientific colleague in Albania, the aspiring graduate student in Kenya, the young doctor in Chile, and the next James Watson or Alexander Fleming from Vietnam can all have unfettered, fee-free, full access to your Open Access article. Why would you want it any other way?

I have a personal reason for supporting Open Access. I am the 1 in 10 Americans born not in the United States but overseas in a developing country. Thirty-five years ago, my father was repeatedly rebuffed in his attempts to secure a visa to enter the United States to study at a graduate school. Had he not persevered, I might today be a frustrated scientist in a developing country unable to pay the subscription fees needed to read papers published in *Nature*, *Cell *or *Virology*. As science and societies move increasingly toward globalization, I am convinced that we all have a responsibility to work towards a knowledge access model that transcends professional classifications, national boundaries, and accidents of birth. As an American, I am confronted by the stark realization that in the just passed year, 2005, 47% of the US national debt was held by foreigners (a large portion by developing nations), and that the American economy stays afloat from an annual inflow of $216 billion from emerging markets (Barron's, March 26, 2006). Hence, should it not be viewed as simple fair reciprocity that American scientists support Open Access as a small gesture of give-back to the rest of the world?

## *Retrovirology's *progress

Having stated the above, I don't believe that it is just altruism which should guide authors to support Open Access. A recently published study in PLOS Biology [1] showed the tangible benefit of Open Access publishing. That study clearly documented Open Access papers to be cited more quickly and more frequently than non-Open Access papers published in the same journal. Hence, it is perhaps not surprising that some long standing traditionally subscription-based journals such as *Nucleic Acids Research *and *Journal of Clinical Investigation *have adopted the completely Open Access model as their new way of publishing.

**Figure 1:**
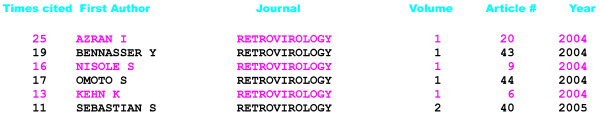
Examples of *Retrovirology *articles that have been cited frequently within the first two years. Data are from ISI's Web of Science.

**Figure 2:**
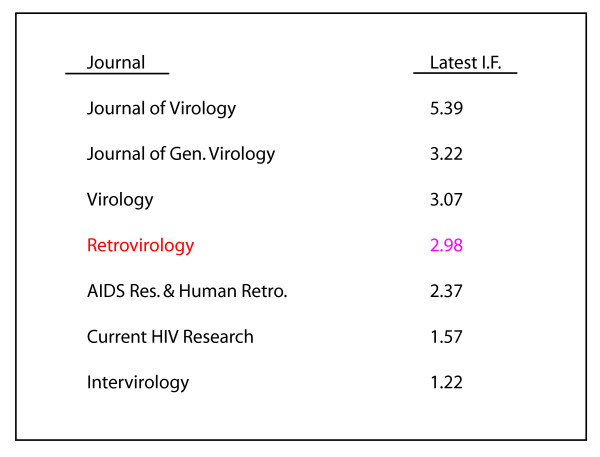
Comparison of *Retrovirology's *preliminary impact factor with selected journals that publish retrovirus research papers.

*Retrovirology's *experience is consistent with the recent report [1]. First, *Retrovirology's *Open Access format unquestionably attracts an impressively large readership. For instance, the *Journal of Molecular Biology*, a leading subscription journal, advertises as having been downloaded "nearly 750,000" times in a single year (2002). *Retrovirology *is a much smaller journal with perhaps 20 times fewer published articles each year; and yet, *Retrovirology *is accessed an average of 1,700 times a day or over 600,000 times a year. I attribute this popularity to our Open Access(ibility). Second, we see a good correlation between the number of times that an article is read and the frequency that it is cited in the literature. This has been verified by access statistics from several of *Retrovirology's *already frequently cited papers (Fig. 1) [2-7]. Finally, my impression is that Open Access has helped *Retrovirology *achieve rapid name recognition and a respectable preliminary Impact Factor number of nearly 3 (i.e. 2.98) just after our very first year of publishing. *Retrovirology's *current impact factor compares very favorably with those of *Virology *and *Journal of General Virology *(Fig. 2).

An impact factor of 3 at age 2 is a good start for *Retrovirology*. Nonetheless, I remain eager to receive your input as to how *Retrovirology *can further improve and do better.

## References

[B1] Eysenbach G (2006). Citation advantage of open access articles. PLoS Biol.

[B2] Azran I, Schavinsky-Khrapunsky Y, Aboud M (2004). Role of Tax protein in human T-cell leukemia virus type-I leukemogenicity. Retrovirology.

[B3] Bennasser Y, Le SY, Yeung ML, Jeang KT (2004). HIV-1 encoded candidate micro-RNAs and their cellular targets. Retrovirology.

[B4] Nisole S, Saib A (2004). Early steps of retrovirus replicative cycle. Retrovirology.

[B5] Omoto S, Ito M, Tsutsumi Y, Ichikawa Y, Okuyama H, Brisibe EA, Saksena NK, Fujii YR (2004). HIV-1 nef suppression by virally encoded microRNA. Retrovirology.

[B6] Kehn K, Deng L, de la Fuente C, Strouss K, Wu K, Maddukuri A, Baylor S, Rufner R, Pumfery A, Bottazzi ME, Kashanchi F (2004). The role of cyclin D2 and p21/waf1 in human T-cell leukemia virus type 1 infected cells. Retrovirology.

[B7] Sebastian S, Luban J (2005). TRIM5alpha selectively binds a restriction-sensitive retroviral capsid. Retrovirology.

